# Robust DNA repair in PAXX‐deficient mammalian cells

**DOI:** 10.1002/2211-5463.12380

**Published:** 2018-02-07

**Authors:** Alisa Dewan, Mengtan Xing, Marie Benner Lundbæk, Raquel Gago‐Fuentes, Carole Beck, Per Arne Aas, Nina‐Beate Liabakk, Siri Sæterstad, Khac Thanh Phong Chau, Bodil Merete Kavli, Valentyn Oksenych

**Affiliations:** ^1^ Department of Clinical and Molecular Medicine (IKOM) Norwegian University of Science and Technology Trondheim Norway; ^2^ St. Olavs Hospital Trondheim University Hospital, Clinic of Medicine Norway; ^3^ Present address: Centre for Immune Regulation and Department of Immunology University of Oslo and Oslo University Hospital‐Rikshospitalet Oslo Norway; ^4^ Present address: KG Jebsen Coeliac Disease Research Centre University of Oslo Oslo Norway

**Keywords:** CH12F3, class switch recombination, etoposide, HAP1, IgA, zeocin

## Abstract

To ensure genome stability, mammalian cells employ several DNA repair pathways. Nonhomologous DNA end joining (NHEJ) is the DNA repair process that fixes double‐strand breaks throughout the cell cycle. NHEJ is involved in the development of B and T lymphocytes through its function in V(D)J recombination and class switch recombination (CSR). NHEJ consists of several core and accessory factors, including Ku70, Ku80, XRCC4, DNA ligase 4, DNA‐PKcs, Artemis, and XLF. Paralog of XRCC4 and XLF (PAXX) is the recently described accessory NHEJ factor that structurally resembles XRCC4 and XLF and interacts with Ku70/Ku80. To determine the physiological role of PAXX in mammalian cells, we purchased and characterized a set of custom‐generated and commercially available NHEJ‐deficient human haploid HAP1 cells, *PAXX* ^*Δ*^ *, XRCC4* ^*Δ*^, and *XLF* ^*Δ*^. In our studies, HAP1 *PAXX* ^*Δ*^ cells demonstrated modest sensitivity to DNA damage, which was comparable to wild‐type controls. By contrast, *XRCC4* ^*Δ*^ and *XLF* ^*Δ*^ HAP1 cells possessed significant DNA repair defects measured as sensitivity to double‐strand break inducing agents and chromosomal breaks. To investigate the role of PAXX in CSR, we generated and characterized *Paxx* ^−/−^ and *Aid* ^*−/−*^ murine lymphoid CH12F3 cells. CSR to IgA was nearly at wild‐type levels in the *Paxx* ^−/−^ cells and completely ablated in the absence of activation‐induced cytidine deaminase (AID). In addition, *Paxx* ^−/−^ CH12F3 cells were hypersensitive to zeocin when compared to wild‐type controls. We concluded that *Paxx*‐deficient mammalian cells maintain robust NHEJ and CSR.

**Abbreviations**
53BP1: p53‐binding protein 1
AID: activation‐induced cytidine deaminase
ATM: ataxia telangiectasia mutated
CSR: class switch recombination
DNA‐PKcs: DNA‐dependent protein kinase, catalytic subunit
DSB: DNA double‐strand break
HRP: horseradish peroxidase
Ig: immunoglobulin
IL: interleukin
Ku: Ku70/Ku80 heterodimer
LPS: lipopolysaccharide
NHEJ: nonhomologous end joining
PAXX: paralog of XRCC4 and XLF
RAG: recombination activating genes 1 and 2
SCID: severe combined immunodeficiency
T‐FISH: telomere fluorescence *in situ* hybridization
UNG: uracil N‐glycosylase
XLF: XRCC4‐like factor
XLS: XRCC4‐like small protein
XRCC4: X‐ray cross‐complementing protein 4

Nonhomologous DNA end joining (NHEJ) is a molecular pathway that recognizes and fixes DNA double‐strand breaks (DSBs) throughout the cell cycle. NHEJ is the key DNA repair pathway involved in B‐ and T‐lymphocyte development through V(D)J recombination, and B‐cell specialization via class switch recombination (CSR) 1. Core NHEJ factors include Ku70, Ku80, XRCC4, and DNA ligase 4. Deficiency in core factors nearly completely abrogates classical NHEJ, whereas alternative end joining is still possible 2. The Ku70/Ku80 heterodimer (Ku) interacts with downstream NHEJ factors. Among others, Ku interacts with XRCC4‐like factor (XLF, or Cernunnos, or NHEJ1), paralog of XRCC4 and XLF (PAXX, or XLS, or C9orf142), and DNA‐dependent protein kinase, catalytic subunit (DNA‐PKcs). Both XLF 3, 4 and PAXX 5, 6, 7 were discovered due to their similarity with XRCC4. While genetic inactivation of *Xrcc4* completely abrogates classical NHEJ and is embryonic lethal in mice 8, inactivation of *Xlf* or *Paxx* has only modest or no effect on mouse development, due to complex functional redundancy in the DNA repair pathway 9. XLF functionally overlaps with PAXX, which is demonstrated in both cell lines and mouse models. For example, combined inactivation of *Xlf* and *Paxx* in mice leads to synthetic lethality 10, 11. In addition, functional overlap between XLF and PAXX in DNA repair was demonstrated using chicken DT40 cell lines 7, and in the NHEJ‐dependent V(D)J recombination using murine pro‐B cells 11, 12, 13, 14. Furthermore, XLF functionally overlaps with DNA damage response factors, protein kinases ATM and DNA‐PKcs, and their substrates, H2AX and 53BP1 15, 16, 17, 18, 19. Currently, the role of PAXX in DSB response, particularly in human cells, is an unsolved question, and it attracted attention of several research groups worldwide.

Class switch recombination is a process in which mature B lymphocytes edit their immunoglobulin heavy‐chain genes in response to antigen stimuli, leading to production of new antibody isotypes with altered downstream effector functions in the immune response. CSR is initiated by transcription‐dependent recruitment of activation‐induced cytidine deaminase (AID) to the cytosine‐rich switch regions of immunoglobulin genes. Upon AID‐induced deamination, cytosines in DNA repair are converted to deoxyuracils, which are then removed by the uracil DNA N‐glycosylase (UNG). Deamination by AID, uracil excision by UNG, and strand incision by AP endonuclease occur simultaneously on both strands, thus leading to DSBs which are repaired by NHEJ 20, 21. Deficiency in core XRCC4 or accessory XLF factors, respectively, leads to a twofold to threefold reduction in CSR 22, 23. However, the role of recently described accessory NHEJ factors in CSR is not determined yet and is an open question.

Here, using a CRISPR/Cas9 approach, we obtained *PAXX*‐deficient human HAP1 cells (*PAXX* ^*Δ*^ *,* Horizon Discovery), generated murine lymphoid CH12F3 cells (*Paxx* ^−/−^) and analyzed several aspects related to DNA repair efficiency of these cell lines, including proliferation, sensitivity to DNA‐damaging chemicals, genomic stability, and CSR. We found that *PAXX* ^*Δ*^ HAP1 cells are not hypersensitive to etoposide and zeocin and do not possess increased levels of genomic instability when compared to WT parental line. Moreover, *Paxx* ^−/−^ CH12F3 cells demonstrated ability to perform robust CSR to IgA when compared to WT and *Aid* ^−/−^ controls.

## Materials and methods

### HAP1 cells

All HAP1 cells (Horizon Discovery) were made commercially available and handled according to the manufacturer's instructions: *XRCC4* ^*Δ*^ (HZGHC000428c019), *XLF* ^*Δ*^ (*NHEJ1* ^*Δ*^, HZGHC000426c004, and HZGHC000426c017), and *PAXX* ^*Δ*^ (*C9orf142* ^*Δ*^, HZGHC004376c005, and HZGHC004376c006).

### Antibodies

For western blot (WB), we used the following antibodies: rabbit polyclonal anti‐PAXX/C9orf142 (NovusBio, Littleton, CO, USA; NBP1‐94172, dilution 1 : 1000); rabbit polyclonal anti‐XLF (Bethyl, A300‐730A, 1 : 2000); swine polyclonal anti‐rabbit Ig‐HRP (Dako antibodies, Dako, Glostrup, Denmark; #P0399, 1 : 5000); mouse monoclonal anti‐XRCC4 (NovusBio, NBP1‐48053, 1 : 2000); mouse monoclonal anti‐β‐actin (Abcam, Cambridge, UK; ab8226, 1 : 2000); rabbit polyclonal anti‐mouse Ig‐HRP (Dako antibodies, #P0260, 1 : 5000); goat polyclonal anti‐mouse Ig‐HRP (Dako antibodies, #P0447, 1 : 5000); rat monoclonal anti‐AID (Active Motif, Carlsbad, CA, USA; #39886, 1 : 500) and secondary anti‐rat IgG, HRP‐linked (Cell signaling #7077, Cell Signaling, Leiden, The Netherlands; 1 : 5000); rabbit polyclonal anti‐UNG (custom‐made against murine UNG, 2 μg·mL^−1^); and IRDye 800CW anti‐rabbit IgG (Licor, Lincoln, NE, USA; #925‐32211, 1 : 15 000).

### Proliferation assay

HAP1 cells were plated 5 × 10^4^ cells·mL^−1^ in triplicate in 6‐well plates and counted every 24 hours using Countess™ Automated Cell Counter (Invitrogen, Carlsbad, CA, USA) with Trypan blue stain (Invitrogen) and bright‐field detection. CH12F3 cells were plated 5 × 10^4^ cells·mL^−1^ in triplicate in 6‐well plates and counted at 24 hours and every 12 hours until 60 hours as described for HAP1.

### Cell survival assay

HAP1 cell sensitivity to DSB‐inducing agents was tested by colony formation assay. Cells were counted, and 100 cells were plated in 2 mL of medium per well, in 6‐well plates on day 0 in triplicates. Etoposide (Sigma‐Aldrich, St. Louis, MO, USA; #E1383) or zeocin (InvivoGen, #15A27‐MM) was added on day 1 at indicated concentrations, and mock‐treated control was used as a reference. On day 10, medium was removed and colonies were stained with 1 mL per well 0.5% crystal violet solution (0.5 g crystal violet powder, 25 mL methanol, 75 mL dH_2_O) for 15 minutes with perturbation 24, 25. After staining crystal violet solution was removed, plates were washed with tap water and colonies were counted. CH12F3 sensitivity to zeocin was assayed with PrestoBlue (ThermoFischer, Waltham, MA, USA), according to the manufacturer's instructions

### Telomeric *in situ* hybridization

Telomeric *in situ* hybridization (T‐FISH) was performed as previously described 16, 17, 18. Metaphase images were captured using a Zeiss TRIF3 microscope equipped with a CCD camera and a 100× objective lens.

### Generation of *Paxx* ^*−/−*^ and *Aid* ^*−/−*^ CH12F3 cell lines

All oligonucleotides corresponding to sgRNAs were cloned into the plasmid vector LentiCRISPR v2 (Addgene plasmid #52961). The following sgRNAs were used to target exon 2 of the *Paxx* gene: TGACGGACGCCGCCGAGCTC, TCTCGCCTGACAGCCTGGCG, and CTCGGCGGCGTCCGTCACAC, using the protocol described in Ref. 26. Upon lentiviral‐mediated transduction of parental WT CH12F3, the cells were subcloned and up to 200 clones from each of the three sgRNAs were screened by WB and cells lacking PAXX signal were kept for experiments. Mock‐treated and parental WT CH12F3 cells were used as control. The *Aid* ^−/−^ CH12F3 cells were obtained by targeting exon 2 of the *Aid* (*Aicda)* gene with AGGGACGGCATGAGACCTAC sgRNA. The cells were subcloned, stimulated to CSR, and screened for AID expression using WB. The cells lacking AID signal detected by WB were verified by DNA sequencing and functional assay (CSR).

### CSR to IgA

Class switch recombination to IgA was induced as previously described 2, 27.

## Results

### Generation and characterization of *PAXX* ^*Δ*^ *, XLF* ^*Δ*^, and *XRCC4* ^*Δ*^ HAP1 cells

**Figure 1:**
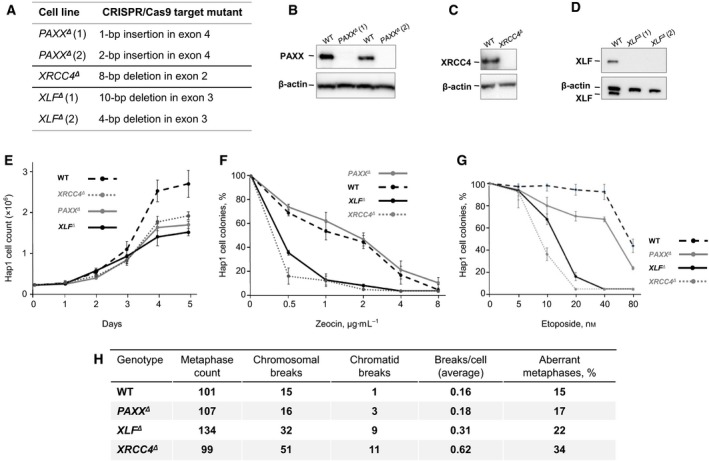
DNA repair efficiency in *PAXX^Δ^, XLF^Δ^* and *XRCC4^Δ^* HAP1 cells. (A) NHEJ‐deficient HAP1 cells were produced by *Horizon Discovery*. Two *PAXX^Δ^* lines carry either 1bp insertion or 2bp deletion in exon 4. *XRCC4^Δ^* line carries 8bp deletion in exon 2. Two *XLF^Δ^* lines carry either 10bp or 4bp deletion in exon 3. (B, C, D) WB assay detected no PAXX protein in *PAXX^Δ^* cells (B), no XRCC4 in *XRCC4^Δ^* cells (C) and no XLF in *XLF^Δ^* cells (D). (E) Proliferation of HAP1 cells of indicated genotypes. We present summary of three experiments, data from two *PAXX^Δ^* and two *XLF^Δ^* lines were pooled. Error bars are S.D. (F, G) Sensitivity of HAP1 cells of indicated genotypes to double‐strand break‐inducing agents Zeocin (F) and Etoposide (G). Numbers represent count of colonies normalized to mock‐treated controls. Four experiments were performed. Data from two *PAXX^Δ^* and two *XLF^Δ^* lines were pooled. Error bars are S.D. (H) Genomic instability in HAP1 cells of indicated genotypes. Count of chromosomal breaks and chromatid breaks; average breaks per cell are shown for reference.

Three XRCC4‐like NHEJ factors have been described in DSB repair: PAXX, XLF, and XRCC4. To characterize the sensitivity of DSB repair in human cells deficient in these factors, we obtained knockout HAP1 cells (Materials and methods). These cells were produced using the CRISPR/Cas9 gene‐editing approach, and the genetic changes were sequence‐verified by Horizon Discovery. In our studies, we used two *PAXX* ^*Δ*^, two *XLF* ^*Δ*^, and one *XRCC4* ^*Δ*^ HAP1 cell lines carrying indel mutations (Fig. 1A and Materials and methods). We verified the absence of corresponding proteins by WB. No band corresponding to PAXX was detected in *PAXX* ^*Δ*^, and no XLF band was observed in *XLF* ^*Δ*^ and no XRCC4 in *XRCC4* ^*Δ*^ cells (Fig. 1B–D). All *PAXX* ^*Δ*^, *XLF* ^*Δ*^, and *XRCC4* ^*Δ*^ HAP1 cells in our experiments proliferated slower than WT parental controls (Fig. 1E), in line with previously observed cell growth defect in *PAXX* ^−/−^, *XLF* ^−/−^, and *XRCC4* ^−/−^ DT40 cell lines 7.

### Sensitivity of *PAXX* ^*Δ*^, *XLF* ^*Δ*^, and *XRCC4* ^*Δ*^ HAP1 cells to DNA‐damaging agents

A number of anticancer agents are used to induce DSBs. To determine whether our knockout HAP1 cells possess hypersensitivity to DSBs when compared to WT parental cells, we treated the cells with zeocin and etoposide, respectively. Both *XLF* ^*Δ*^ and *XRCC4* ^*Δ*^ cells were hypersensitive to zeocin at concentrations ranging from 0.5 to 4 μg·mL^−1^ while sensitivity of *PAXX* ^*Δ*^ cells was not different from WT control. Higher doses of zeocin were lethal for all the cell lines (Fig. 1F). Similarly, *XLF* ^*Δ*^ and *XRCC4* ^*Δ*^ HAP1 cells were hypersensitive to etoposide at concentrations ranging from 5 to 80 nM while *PAXX* ^*Δ*^ cells were modestly sensitive to etoposide (Fig. 1G).

### Genomic instability in *PAXX* ^*Δ*^, *XLF* ^*Δ*^, and *XRCC4* ^*Δ*^ HAP1 cells

Inactivation of *NHEJ* genes usually leads to accumulation of translocations, chromatin, and chromosomal breaks 1. To determine levels of genomic instability in *PAXX* ^*Δ*^ *, XLF* ^*Δ*^, and *XRCC4* ^*Δ*^ HAP1 cells, we performed T‐FISH analysis. We observed relatively high background levels of genomic instability (measured as chromosomal and chromatid breaks) in WT HAP1 cells with 0.16 break per cell on average, which is higher than we previously observed in NHEJ‐proficient murine cells (0.00–0.10 breaks per cell, 16, 17, 18) (Fig. 1H). Genomic instability nearly did not increase in *PAXX* ^*Δ*^ cells with 0.18 breaks per cell. Furthermore, we observed 0.31 breaks per cell in *XLF* ^*Δ*^ and 0.62 breaks per cell in *XRCC4* ^*Δ*^ HAP1 cells. This finding suggests that XRCC4 and XLF have major role to maintain genomic stability in HAP1 cells, while PAXX has no obvious function in this process.

### Class Switch Recombination to IgA in *Paxx* ^*−/−*^ and *Aid* ^*−/−*^ CH12F3 cells

**Figure 2:**
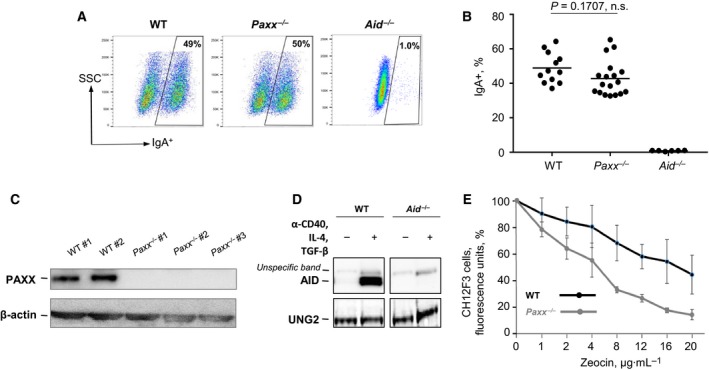
Robust CSR to IgA^+^ in *Paxx* ^−/−^ CH12F3 cells. (A) Example of CH12F3 FACS plot of indicated genotypes 4 days after stimulation with anti‐CD40, IL‐4, and TGF‐β. (B) CSR summary of six experiments of two WT (pooled) and three *Paxx* ^−/−^ (pooled) CH12F3 cells. *Aid* ^−/−^ CH12F3 cells were used to establish background levels of experiments. Multiple comparisons between groups were performed using GraphPad Prism 7.03 (La Jolla, CA, USA) and one‐way ANOVA. (C) Detection of PAXX protein by WB in WT and *Paxx* ^−/−^ CH12F3 cells. β‐Actin was used as a loading control. (D) Detection of AID protein using WB. UNG2 was used as a loading control. ‘**+**’ indicates cells stimulated with anti‐CD40, IL‐4 and TGF‐β. ‘−’ indicates unstimulated cells. AID expression was induced and detected in stimulated WT cells; AID band was absent before and after stimulation of *Aid* ^−/−^ cells. (E) Sensitivity of WT and *Paxx* ^−/−^ cells to DSB‐inducing agent zeocin. Numbers represent cell count normalized to mock‐treated controls. Three experiments with three independent clones were performed. Error bars represent SD.

Most of the core and accessory NHEJ factors are required for efficient CSR 1. To elucidate the role of PAXX in CSR, we applied CRISPR/Cas9 to generate frame shift in exon 2 of *Paxx* gene in murine B‐cell line CH12F3 (Materials and methods). We screened individual clones by WB and selected three *Paxx* ^−/−^ subclones originating from independent sgRNAs. In six experiments, *Paxx* ^−/−^ and WT cells switched to IgA with certain variation between each experiment and cell line (Fig. 2A–C). On average, at day 4 after the CSR stimulation, 43% of *Paxx* ^−/−^ cells expressed *IgA*, which was similar or lower when compared to WT controls, on average 49% of *IgA* on day 4, *p* = 0.1707 (Fig. 2B). To establish background levels in our CSR experiments, we generated and used *Aid* ^−/−^ CH12F3 cells by targeting exon 2 of *Aid* gene by CRISPR/Cas9 approach. Background levels of IgA detected in *Aid* ^−/−^ cells varied from 0.4% to 1.0% with average 0.8%, which was significantly lower when compared to both *Paxx* ^−/−^ and WT lines (*p* < 0.0001, ****; Fig. 2A, B, D). These experiments are in line with previously published observations that inactivation of *Paxx* in CH12F3 cell lines has no effect on the CSR to IgA 14, and Paxx null mice possess normal CSR to various isotypes including the IgG1, IgG2, and IgG3 10, 11.

### Sensitivity of *Paxx* ^*−/−*^ CH12F3 cells to DSBs

To examine whether PAXX is required for DSBs repair in murine lymphoid cells, we treated WT and *Paxx* ^−/−^ CH12F3 lines with the DSB‐inducing agent zeocin. Contrary to HAP1, *Paxx* ^−/−^ CH12F3 cells showed higher levels of sensitivity to zeocin when compared to parental WT lines (Fig. 2E). In addition, we examined proliferation of WT and three *Paxx* ^−/−^ CH12F3 lines by counting cells every 12 hours up to 72 hours. In three independent experiments, all the clones proliferated with similar speed and there was no difference in the cell cycle distribution (not shown). We concluded that PAXX is required for optimal DNA repair of zeocin‐induced DSBs in murine cell lines, although it is dispensable for CSR.

## Discussion

Here, we purchased and characterized *PAXX‐*deficient human fibroblast‐like HAP1 cells (Horizon Discovery) and generated murine lymphoid CH12F3 cell lines at our own laboratory. We concluded that PAXX is dispensable for CSR and has a modest impact on cell resistance to DSB‐inducing agents such as etoposide and zeocin.

Previous studies had controversial conclusions on the role of PAXX in DNA repair. For instance, significantly increased sensitivity to ionizing radiation was observed in *Paxx*‐knockout murine pro‐B cells 11, 12, mouse embryonic fibroblasts 11, chicken DT40 cells 7, and human *PAXX*‐knockdown U2OS cells 5, 6. *PAXX* knockout, however, resulted in no change in sensitivity to DSB‐inducing agent zeocin in human embryonic kidney cells 28 and doxorubicin in chicken DT40 cells 7. We explain this discrepancy by two main reasons. First, cell lines from different species were used in the studies, including chicken, mouse, and human. Second, distinct ways to induce DSBs were selected, including ionizing radiation, zeocin, doxorubicin, and etoposide. In addition, RAG‐ and AID‐/UNG‐mediated semiphysiological systems were used to study effect of PAXX deficiency on V(D)J recombination and CSR. In addition, slower proliferation of *PAXX‐*,* XLF‐,* and *XRCC4‐*deficient cells when compared to parental cell lines might be a specific feature of HAP1 and one may speculate that it depends on the transformation status or cell line origin. Similar proliferation defect was observed in the NHEJ‐deficient DT40 cells in our previous studies 7. Although in the current study PAXX has no or minor effect on the NHEJ measured as the CSR or indirectly assayed as the sensitivity to DSB‐inducing agents, genetic models used by us and our colleagues revealed functional overlap of PAXX with XLF [7, 10, 11, 12, 14]. It is possible that PAXX complements or substitutes other DNA repair factors; for example, accessory NHEJ‐ or ATM‐dependent DNA damage signaling proteins, and complex genetic models are required to identify its functions.

## Author contributions

AD, MX, RGF, CB, NL, SS, KTPH, and VO planned and performed experiments and interpreted results. BK, ML, and PAA provided *Aid* ^−/−^ CH12F3 cell lines and expertise in CRISPR/Cas9 experiments. VO wrote the manuscript.
