# Corrigendum to: Robust DNA repair in PAXX‐deficient mammalian cells

**DOI:** 10.1002/2211-5463.12776

**Published:** 2020-01-06

In Figure 1B of the paper by Dewan *et al*. 1, the western blot lanes were incorrectly labelled. From left to right, the lanes should have been labelled as WT, *PAXX^∆^*, *DNA‐PKcs^∆^* and *PAXX^∆^ DNA‐PKcs^∆^*. This does not affect the conclusions of this paper. The corrected version of Figure 1 and its legend are provided here:

**Figure 1:**
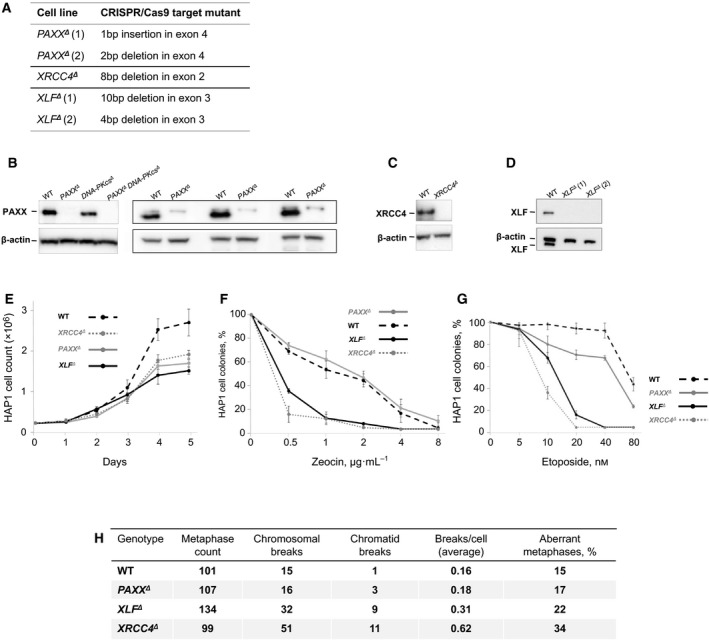
DNA repair efficiency in *PAXX^∆^*, *XLF^∆^* and *XRCC4^∆^* HAP1 cells. (A) NHEJ‐deficient HAP1 cells were produced by Horizon Discovery. Two *PAXX^∆^* lines carry either 1‐bp insertion or 2‐bp deletion in exon 4. *XRCC4^∆^* line carries 8‐bp deletion in exon 2. Two *XLF^∆^* lines carry either 10‐bp or 4‐bp deletion in exon 3. (B, C, D) WB assay detected no PAXX protein in *PAXX^∆^* and *PAXX^∆^* *DNA‐PKcs^∆^* cells (B), no XRCC4 in *XRCC4^∆^* cells (C) and no XLF in *XLF^∆^* cells (D). (E) Proliferation of HAP1 cells of indicated genotypes. We present summary of three experiments; data from two *PAXX^∆^* and two *XLF^∆^* lines were pooled. Error bars are SD. (F, G) Sensitivity of HAP1 cells of indicated genotypes to double‐strand break‐inducing agents zeocin (F) and etoposide (G). Numbers represent count of colonies normalized to mock‐treated controls. Four experiments were performed. Data from two *PAXX^∆^* and two *XLF^∆^* lines were pooled. Error bars are SD. (H) Genomic instability in HAP1 cells of indicated genotypes. Count of chromosomal breaks and chromatid breaks; average breaks per cell are shown for reference.
